# Occupation, industry, and the risk of prostate cancer: a case-control study in Montréal, Canada

**DOI:** 10.1186/s12940-016-0185-1

**Published:** 2016-10-21

**Authors:** Jean-François Sauvé, Jérôme Lavoué, Marie-Élise Parent

**Affiliations:** 1 School of Public Health, Department of environmental and occupational health, Université de Montréal, C.P. 6128 Succursale Centre-Ville, Montréal, Québec H3C 3J7 Canada; 2 Centre de recherche du CHUM, 850 rue Saint-Denis, Montréal, Québec H2X 0A9 Canada; 3 Epidemiology and Biostatistics UnitEpidemiology and Biostatistics Unit, INRS-Institut Armand-Frappier, Institut national de la recherche scientifique, Université du Québec, 531 Boul. des Prairies, Laval, Québec H7V 1B7 Canada; 4 School of Public Health, Department of Social and Preventive Medicine, Université de Montréal, C.P. 6128 Succursale Centre-Ville, Montréal, Québec H3C 3J7 Canada

**Keywords:** Prostate cancer, Occupation, Industry

## Abstract

**Background:**

Age, family history and ancestry are the only recognized risk factors for prostate cancer (PCa) but a role for environmental factors is suspected. Due to the lack of knowledge on the etiological factors for PCa, studies that are both hypothesis-generating and confirmatory are still needed. This study explores relationships between employment, by occupation and industry, and PCa risk.

**Methods:**

Cases were 1937 men aged ≤75 years with incident PCa diagnosed across Montreal French hospitals in 2005-2009. Controls were 1994 men recruited concurrently from electoral lists of French-speaking Montreal residents, frequency-matched to cases by age. In-person interviews elicited occupational histories. Unconditional logistic regression estimated odds ratios (OR) and 95 % confidence intervals (CI) for the association between employment across 696 occupations and 613 industries and PCa risk, adjusting for potential confounders. Multinomial logistic models assessed risks by PCa grade. Semi-Bayes (SB) adjustment accounted for the large number of associations evaluated.

**Results:**

Consistently positive associations—and generally robust to SB adjustment—were found for occupations in forestry and logging (OR 1.9, 95 % CI: 1.2–3.0), social sciences (OR 1.6, 95 % CI: 1.1–2.2) and for police officers and detectives (OR: 1.8, 95 % CI 1.1–2.9). Occupations where elevated risk of high grade PCa was found included gasoline station attendants (OR 4.3, 95 % CI 1.8–10.4) and textile processing occupations (OR 1.8, 95 % CI 1.1–3.2). Aside from logging, industries with elevated PCa risk included provincial government and financial institutions. Occupations with reduced risk included farmers (OR 0.6, 95 % CI 0.4–1.0) and aircraft maintenance workers (OR 0.1, 95 % CI 0.0–0.7).

**Conclusions:**

Excess PCa risks were observed across several occupations, including predominantly white collar workers. Further analyses will focus on specific occupational exposures.

**Electronic supplementary material:**

The online version of this article (doi:10.1186/s12940-016-0185-1) contains supplementary material, which is available to authorized users.

## Background

Prostate cancer (PCa) is the most frequently diagnosed cancer among Canadian men, with over 20,000 new cases and 4000 deaths per year [1]. PCa is also the most frequently diagnosed cancer in the United States [2] and the second worldwide after lung cancer [3]. Recognized risk factors for PCa are limited to age, first-degree family history of PCa and ethnicity [4]. The influence of environmental and lifestyle factors in the etiology of this disease has long been suggested, including factors from the work environment, the latter having been reviewed by Parent and Siemiatycki [5] and more recently by Doolan, et al. [6]. Notably, the International Agency for Research on Cancer (IARC) considers that limited evidence exists for arsenic and inorganic compounds, cadmium and cadmium compounds, malathion and X and gamma radiation, as well as employment in rubber manufacturing [7].

Many studies have focused on farming and pesticide application as elevated PCa incidence and mortality among farmers have been documented since the 1980s. Studies such as the Agricultural Health Study (AHS) in the United States have provided some clues as to agents that might be implicated, mainly organochlorines and organophosphates. More research will help characterize the potential interplay between these exposures and genetic factors in PCa etiology [8]. Other occupations that have been associated with elevated risks of PCa include firefighters [9], aviation-related [10, 11], administrative and managerial [12, 13], metalworking [5] and rubber production [14] occupations. However, results from occupation-based studies have generally been inconsistent or inconclusive. Very few of them have taken into account PCa aggressiveness [15–18]. For instance, preliminary results from an American case-control study [18] found relationships between employment in truck driving or gardening occupations and aggressive PCa relative to lower PCa grade. Associations between aggressive PCa and selected organochlorine and organophosphate pesticides were also found in the AHS [19] which, in the case of Diazinon, was not apparent when looking at total PCa [20].

This study aims at further exploring potential associations between employment across a wide range of occupations and industries and PCa risk, both overall and stratified by PCa grade, using data from a large, population-based case-control study conducted in Montreal, Canada.

## Methods

### Study population

The Prostate Cancer and Environment Study (PROtEuS) is a population-based case-control study conducted in Greater Montreal, Canada, initiated to explore the role of lifestyle, environmental and occupational factors in PCa etiology. The study has been described in detail previously [21]. Briefly, eligible subjects were men aged ≤75 years at diagnosis or recruitment, Canadian citizens, registered on the permanent electoral list and residing in one of the 39 electoral districts of the greater Montreal area.

Cases were all patients newly diagnosed with primary histologically confirmed PCa from September 2005 to December 2009, actively ascertained from pathology departments across the main Montreal hospitals serving the French-speaking population. These represent over 80 % of all new cases in the area according to registry information. Concurrently, population controls were randomly selected from the electoral list of French-speaking men, frequency-matched to cases by 5-year age intervals. Participation rates for eligible cases and controls were 79.4 % and 55.5 %, respectively. Ethics boards of all participating institutions approved the study; all subjects provided written informed consent.

### Data collection

Between 2006 and 2012, in-person interviews were conducted by trained interviewers, mainly at the subjects’ homes, to collect a complete occupational history covering each job held for at least 1 year during their career, including first and last year of employment, company name and main tasks performed. Subjects also provided information on a variety of socio-demographic characteristics, anthropometric, lifestyle and environmental factors, as well as medical and residential histories. Gleason scores were extracted from cases’ biopsy pathology reports to define PCa grades.

### Coding of occupation and industry titles

A team of industrial hygienists reviewed the occupational histories (blind to case/control status) to assign occupation and industry titles for each job. Occupations were coded in the 1971 (updated through 1980) Canadian Classification and Dictionary of Occupations (CCDO) [22] scheme, which has a hierarchical structure featuring 2-digit major groups, 3-digit minor groups, 4-digit unit groups and 7-digit occupations. Industry titles were based on the 1980 Canadian Standardized Industrial Classification (SIC) [23] defined by 2-digit major groups, 3-digit minor groups and 4-digit industry classes.

### Statistical analyses

For each occupation and industry category, unconditional logistic regression models estimated odds ratios (OR) and 95 % confidence intervals (95 % CI) for the risk of PCa according to ever employment and duration of employment (<10 and ≥10 years). For a given occupation or industry category, subjects never employed in that particular occupation or industry represented the reference group. ORs were estimated for each 2, 3, 4 and 7-digit CCDO and 2, 3 and 4-digit SIC categories with at least 10 subjects (including 1 case and 1 control) ever employed.

Models were adjusted for the three recognized risk factors for PCa: age (as a continuous variable), first-degree family history of PCa (yes, no, unknown), ancestry (European, Sub-Saharan African, Asian, Middle Eastern, other (e.g. Hispanic, Aboriginal), unknown) and timing of last PCa screening before diagnosis or interview (≤2 years, >2 years, never screened, unknown). Inclusion of the following additional covariates in the models was tested using a stepwise procedure and Akaike’s Information criterion: Annual household income (<$C30,000, $C30,000–79,999, $C80,000 or more, refusal, unknown), highest level of education attained (primary, secondary/college, university, unknown), self-reported level of physical activity at work and at home (not very active, moderately active, very active), cigarette pack-years (zero, tertiles above zero, unknown), alcohol drink-years (zero, tertiles above zero, unknown) and quartiles of body-mass index (BMI) 2 years before diagnosis or interview. All variables except cigarette pack-years were retained in the final models.

In addition, multinomial logistic regression models were used to evaluate associations according to PCa grade. Low grade was defined by a Gleason score less than 7 or (3 + 4) and high grade by a Gleason score of 8 or higher or (4 + 3) [24]; eight cases were excluded from this analysis due to incomplete information on Gleason scores. The same set of potential confounders retained in the binary logistic regression models was used.

#### Semi-Bayes adjustment for multiple comparisons

The large number of occupations and industries evaluated may lead to a non-negligible amount of false-positive associations being observed due to chance. To identify the more robust estimates, we applied a shrinkage-based method using Semi-Bayes adjustment (SB) [25]. Prior applications of SB methods in job title analyses have included separate adjustment for *a priori* “low” or “high” risk occupations for bladder cancer [26], or accounting for exposures to known lung carcinogens within occupations [27]. In our case, there was no clear a priori expectation of occupations or industries being strongly associated with increased or decreased PCa risk. Therefore, for each combination of cancer grade (all PCa, low or high grade), type of analysis (occupations or industries), and duration (ever/never or duration in years), estimates were shrunk towards a common mean. For each analysis, a prior variance of 0.25 of the ORs on the log scale was used, corresponding to approximately 95 % of the “true” ORs lying between 0.38 and 2.66 (7-fold difference). In addition, while the analysis was restricted to occupations or industries with at least 10 subjects ever employed, the stratification by duration of employment and by cancer grade could result in estimates based on a smaller sample. Therefore, the application of SB adjustment was limited to categories with at least five subjects employed.

Analyses were performed using the R software (version 3.1.0, R Development Core Team, Vienna, Austria).

## Results

The study population comprised 1937 PCa cases and 1994 population controls. Among cases, 524 were classified as having high grade PCa, 1405 low grade and 8 had insufficient information to be classified in either category. Proxy respondents, mainly spouses, completed the interview for 50 cases (3.1 %) and 77 controls (3.9 %). Selected characteristics of cases and controls are presented in Table 1. Controls were slightly older than cases on average at interview (mean of 64.8 years) compared to cases at diagnosis (mean of 63.6 years), reflecting the slightly longer time required to secure interviews with controls. As expected, subjects from Sub-Saharan ancestry and with first-degree family history of PCa were more likely to be cases, while subjects of Asian ancestry were more likely to be controls. The proportion of controls screened at least once by prostatic specific antigen (PSA) and/or digital rectal examination (DRE) in the 2 years preceding the date of interview was relatively high at 76 %. Annual household income, highest level of education, physical activity level, cigarette pack-years and alcohol intake level were fairly similar between cases and controls. There were more controls in the highest quartile of BMI compared to cases. The occupational histories of the 3931 subjects covered 19,373 unique jobs spanning the period 1943–2012. The number of jobs held during lifetime per subject ranged from 0 (2 cases and 2 controls) to 23, with an average of 4.9.

**Table 1:** Selected characteristics of cases and controls

Variable	Controls (*n* = 1994)	Cases		
		All (*n* = 1937)	Low grade^a^ (*n* = 1405)	High grade^b^ (*n* = 524)
Age at diagnosis or interview in years, n (%)				
< 60	446 (22.4)	523 (27.0)	414 (29.5)	107 (20.4)
≥ 60 and <65	450 (22.6)	486 (25.1)	351 (25.0)	131 (25.0)
≥ 65 and <70	522 (26.2)	498 (25.7)	347 (24.7)	150 (28.6)
≥ 70 and ≤75	576 (28.9)	430 (22.2)	293 (20.9)	136 (26.0)
Ancestry, n (%)				
European	1685 (84.5)	1696 (87.6)	1234 (87.8)	455 (86.8)
Sub-Saharan	90 (4.5)	130 (6.7)	99 (7.0)	30 (5.7)
Asian	73 (3.7)	24 (1.2)	15 (1.1)	9 (1.7)
Greater Middle East	99 (5.0)	45 (2.3)	32 (2.3)	13 (2.5)
Other	33 (1.7)	30 (1.5)	18 (1.3)	12 (2.3)
Don’t know	14 (0.7)	12 (0.6)	7 (0.5)	5 (1.0)
First-degree family history of PCa, n (%)				
No	1739 (87.2)	1419 (73.3)	1008 (71.7)	405 (77.3)
Yes	199 (10.0)	452 (23.3)	351 (25.0)	100 (19.1)
Don’t know	56 (2.8)	66 (3.4)	46 (3.3)	19 (3.6)
Date of last screening for PCa before diagnosis or interview, n (%)				
≤ 2 years	1510 (75.7)	1917 (99.0)	1388 (98.8)	521 (99.4)
> 2 years	235 (11.8)	1 (0.1)	1 (0.1)	0 (0.0)
No/Never	191 (9.6)	3 (0.2)	2 (0.1)	1 (0.2)
Don’t know	58 (2.9)	16 (0.8)	14 (1.0)	2 (0.4)
Annual household income in CAD$, n (%)				
< 10,000–29,999	497 (24.9)	490 (25.3)	323 (23.0)	167 (31.9)
30,000–79,999	872 (43.8)	874 (45.1)	640 (45.6)	228 (43.5)
80,000 and more	428 (21.5)	426 (22.0)	343 (24.4)	81 (15.5)
Prefers not to respond	186 (9.3)	132 (6.8)	91 (6.5)	41 (7.8)
Don’t know	9 (0.5)	15 (0.8)	8 (0.6)	7 (1.3)
Highest level of education attained, n (%)				
Primary	428 (21.5)	449 (23.2)	312 (22.2)	136 (26.0)
Secondary/College	953 (47.8)	891 (46.0)	629 (44.8)	259 (49.5)
University	611 (30.7)	592 (30.6)	461 (32.8)	127 (24.3)
Don’t know	1 (0.1)	4 (0.2)	3 (0.2)	1 (0.2)
Self-reported level of physical activity at work or leisure, n (%)				
Not very active	151 (7.6)	127 (6.6)	84 (6.0)	43 (8.2)
Moderately active	753 (37.8)	682 (35.2)	516 (36.7)	160 (30.5)
Very active	1089 (54.6)	1128 (58.2)	805 (57.3)	321 (61.3)
Cigarette pack-years, n (%)				
Zero	544 (27.3)	542 (28.0)	404 (28.8)	136 (26.0)
> 0–15.1	441 (22.1)	498 (25.7)	381 (27.1)	114 (21.8)
> 15.1–39.4	524 (26.3)	420 (21.7)	296 (21.1)	122 (23.3)
> 39.4–223	474 (23.8)	458 (23.6)	313 (22.3)	144 (27.5)
Don’t know	11 (0.6)	19 (1.0)	11 (0.8)	8 (1.5)
Alcohol drink-years, n (%)				
Zero	231 (11.6)	214 (11.0)	157 (11.2)	56 (10.7)
> 0–24.4	575 (28.8)	548 (28.3)	413 (29.4)	133 (25.4)
> 24.4–76	569 (28.5)	553 (28.5)	408 (29.0)	143 (27.3)
> 76–2660	577 (28.9)	545 (28.1)	376 (26.8)	166 (31.7)
Don’t know	42 (2.1)	77 (4.0)	51 (3.6)	26 (5.0)
Body-mass index, 2 years before diagnosis or interview, in kg/m^2^, n (%)				
≤ 24.2	477 (23.9)	502 (25.9)	362 (25.8)	137 (26.2)
> 24.2–26.5	492 (24.7)	512 (26.4)	373 (26.5)	137 (26.2)
> 26.5–29.2	477 (23.9)	479 (24.7)	362 (25.8)	116 (22.2)
> 29.2	535 (26.8)	431 (22.3)	299 (21.3)	130 (24.9)
Don’t know	13 (0.7)	12 (0.6)	9 (0.6)	3 (0.6)

^a^Gleason score ≤6 or 3 + 4

^b^Gleason score ≥8 or 4 + 3

A total of 2993 2, 3, 4 and 7-digit CCDO groups were represented in data, with 696 (23 %) having at least 10 subjects ever employed. The risk of PCa (all PCa and by PCa grade) associated with each of these 696 groups are presented in [Additional file 1: Table S1], while remaining occupations (*n* = 2297) are listed in [Additional file 2 Table S3]. Occupations with a significantly elevated or reduced PCa risk are presented in Table 2 for all PCa, and in Table 3 for high grade PCa.

**Table 2:** Associations between selected occupations and risk of prostate cancer (all cancers)^a^

CCDO code and description	Never	Ever employed			<10 years employed			≥10 years employed		
	Ca/Co	Ca/Co	OR (95 % CI)	OR SB (95 % CI)	Ca/Co	OR (95 % CI)	OR SB (95 % CI)	Ca/Co	OR (95 % CI)	OR SB (95 % CI)
111: Officials and administrators unique to government	1875/1936	62/58	1.2 (0.8–1.8)	1.2 (0.8–1.7)	19/31	0.7 (0.4–1.3)	0.8 (0.5–1.3)	43/27	1.8 (1.0–3.1)^b^	1.6 (0.9–2.5)
1116: Inspectors and regulatory officers, government	1910/1982	27/12	2.7 (1.2–6.2)^b^	1.8 (1.0–3.4)	11/4	2.9 (0.8–10.7)	1.5 (0.7–3.3)	16/8	2.6 (0.9–7.4)	1.6 (0.8–3.2)
1130126: General manager, finance (bank. & finance)	1925/1989	12/5	3.5 (1.0–12.5)^b^	1.6 (0.8–3.5)	5/3	1.9 (0.4–10.2)	1.2 (0.5–2.8)	7/2	6.7 (0.9–48.2)	1.5 (0.6–3.5)
1143: Production management occupations	1911/1954	26/40	0.7 (0.4–1.3)	0.8 (0.5–1.3)	12/12	1.6 (0.6–4.0)	1.3 (0.7–2.5)	14/28	0.5 (0.2–1.0)^b^	0.6 (0.4–1.1)
1171162: Auditor (prof. & tech., n.e.c.)	1908/1975	29/19	1.7 (0.9–3.3)	1.4 (0.8–2.5)	9/11	0.9 (0.3–2.3)	0.9 (0.5–1.9)	20/8	2.9 (1.1–7.3)^b^	1.8 (0.9–3.4)
1179299: Other occupations related to management and administration	1916/1985	21/9	2.8 (1.1–6.9)^b^	1.7 (0.9–3.4)	12/6	2.5 (0.8–7.7)	1.5 (0.7–3.1)	9/3	3.4 (0.7–16.3)	1.4 (0.6–3.3)
21: Occupations in natural sciences, engineering and mathematics	1713/1722	224/272	0.8 (0.7–1.1)	0.8 (0.7–1.1)	85/75	1.1 (0.8–1.6)	1.1 (0.8–1.6)	139/197	0.7 (0.6–0.9)^b^	0.8 (0.6–1.0)^b^
2144: Electrical engineers	1926/1965	11/29	0.4 (0.2–0.8)^b^	0.5 (0.3–1.0)^b^	2/13	0.1 (0.0–0.5)^b^	0.5 (0.2–1.2)	9/16	0.8 (0.3–1.9)	0.9 (0.4–1.7)
2144110: Design and development engineer, electrical and electronic (prof. & tech., n.e.c.)	1934/1987	3/7	0.2 (0.1–1.0)	0.6 (0.3–1.5)	1/5	0.1 (0.0–0.8)^b^	0.7 (0.3–1.6)	2/2	1.5 (0.1–17.6)	
23: Occupations in social sciences and related fields	1809/1914	128/80	1.6 (1.1–2.2)^b^	1.5 (1.1–2.1)^b^	52/30	1.6 (1.0–2.6)	1.4 (0.9–2.3)	76/50	1.6 (1.0–2.5)^b^	1.5 (1.0–2.2)^b^
2391118: Counsellor, educational (educ.)	1918/1988	19/6	3.0 (1.1–8.3)^b^	1.7 (0.9–3.5)	12/4	2.4 (0.8–7.4)	1.4 (0.7–3.0)	7/2	5.7 (0.8–43.4)	1.4 (0.6–3.4)
271: University teaching and related occupations	1852/1933	85/61	1.5 (1.0–2.2)^b^	1.4 (1.0–2.0)	29/20	1.4 (0.7–2.6)	1.3 (0.7–2.2)	56/41	1.5 (1.0–2.4)	1.4 (0.9–2.2)
315: Other occupations in medicine and health	1913/1973	24/21	1.0 (0.5–1.9)	1.0 (0.6–1.7)	19/6	3.2 (1.0–9.8)^b^	1.7 (0.8–3.5)	5/15	0.3 (0.1–0.9)^b^	0.6 (0.3–1.2)
335: Occupations in writing	1919/1962	18/32	0.6 (0.3–1.2)	0.7 (0.4–1.2)	10/12	1.5 (0.5–4.3)	1.2 (0.6–2.5)	8/20	0.3 (0.1–0.8)^b^	0.6 (0.3–1.0)
4171: Receptionists and information clerks	1918/1986	19/8	3.0 (1.1–8.0)^b^	1.7 (0.9–3.5)	19/8	3.0 (1.1–8.0)^b^	1.7 (0.9–3.5)	0/0		
4197: General office clerks	1839/1910	98/84	1.3 (0.9–1.8)	1.2 (0.9–1.7)	81/53	1.6 (1.1–2.3)^b^	1.5 (1.0–2.1)^b^	17/31	0.6 (0.3–1.2)	0.7 (0.4–1.3)
513/514: Sales occupations, commodities	1564/1569	373/425	0.9 (0.7–1.0)	0.9 (0.7–1.0)	198/193	1.0 (0.8–1.3)	1.0 (0.8–1.3)	175/232	0.8 (0.6–0.9)^b^	0.8 (0.6–0.9)^b^
5135182: Salesperson, footwear (ret. Trade)	1936/1985	1/9	0.1 (0.0–0.9)^b^	0.7 (0.3–1.7)	0/7			1/2	0.8 (0.1–8.9)	
5137: Sales clerks, commodities	1833/1908	104/86	1.2 (0.9–1.7)	1.2 (0.9–1.6)	82/58	1.5 (1.0–2.3)^b^	1.5 (1.0–2.1)^b^	22/28	0.7 (0.4–1.2)	0.8 (0.4–1.2)
5137111: Supermarket clerk (ret. Trade)	1900/1968	37/26	1.6 (0.9–2.9)	1.4 (0.9–2.4)	32/18	2.3 (1.2–4.7)^b^	1.8 (1.0–3.1)^b^	5/8	0.5 (0.2–1.7)	0.8 (0.4–1.6)
5145: Service station attendants	1912/1982	25/12	2.3 (1.0–5.1)^b^	1.7 (0.9–3.1)	21/11	2.0 (0.9–4.8)	1.5 (0.8–2.9)	4/1	4.6 (0.5–42.0)	1.3 (0.5–3.2)
5199158: Telephone solicitor (any ind.)	1930/1981	7/13	0.5 (0.2–1.3)	0.7 (0.3–1.4)	3/12	0.2 (0.1–0.9)^b^	0.6 (0.3–1.3)	4/1	2.7 (0.3–24.6)	1.2 (0.5–2.9)
6112: Police officers and detectives, government	1892/1964	45/30	1.8 (1.1–2.9)^b^	1.6 (1.0–2.4)	13/5	3.2 (1.0–10.0)^b^	1.7 (0.8–3.5)	32/25	1.5 (0.8–2.6)	1.4 (0.8–2.2)
6112146: Detective (gov. Serv.)	1925/1987	12/7	2.0 (0.7–5.2)	1.4 (0.7–2.8)	9/1	10.3 (1.3–82.9)^b^	1.5 (0.6–3.8)	3/6	0.5 (0.1–2.3)	0.8 (0.4–1.9)
6112158: Police officer (gov. Serv.)	1899/1972	38/22	2.0 (1.1–3.5)^b^	1.7 (1.0–2.7)^b^	18/7	3.4 (1.3–8.8)^b^	1.9 (0.9–3.7)	20/15	1.4 (0.7–2.9)	1.3 (0.7–2.3)
6117190: Infantry soldier (military)	1877/1898	60/96	0.7 (0.5–1.0)	0.7 (0.5–1.0)	57/95	0.7 (0.5–1.0)^b^	0.7 (0.5–1.0)	3/1	2.4 (0.2–24.2)	
612: Food and beverage preparation and related service occupations	1797/1813	140/181	0.7 (0.6–1.0)^b^	0.8 (0.6–1.0)^b^	77/103	0.7 (0.5–1.0)^b^	0.7 (0.5–1.0)	63/78	0.8 (0.6–1.2)	0.8 (0.6–1.2)
6121130: Short-order cook (cater. & lodg.)	1933/1982	4/12	0.2 (0.1–0.8)^b^	0.6 (0.3–1.3)	2/7	0.3 (0.0–1.6)	0.8 (0.3–1.8)	2/5	0.2 (0.0–1.2)	0.7 (0.3–1.7)
613: Occupations in lodging and other accommodation	1915/1965	22/29	0.7 (0.4–1.2)	0.8 (0.4–1.3)	16/13	1.1 (0.5–2.5)	1.1 (0.6–2.0)	6/16	0.3 (0.1–0.9)^b^	0.6 (0.3–1.2)
6145: Travel and related attendants, except food and beverage	1935/1986	2/8	0.2 (0.0–0.9)^b^	0.6 (0.3–1.5)	1/4	0.2 (0.0–2.4)	0.8 (0.3–2.0)	1/4	0.1 (0.0–1.5)	0.8 (0.3–1.9)
619: Other service occupations	1759/1799	178/195	0.8 (0.6–1.0)^b^	0.8 (0.6–1.0)^b^	117/113	0.8 (0.6–1.1)	0.8 (0.6–1.1)	61/82	0.7 (0.5–1.0)	0.7 (0.5–1.0)
6191126: Hospital cleaner (misc. Serv.)	1921/1973	16/21	0.6 (0.3–1.2)	0.7 (0.4–1.3)	8/15	0.3 (0.1–0.9)^b^	0.6 (0.3–1.1)	8/6	1.4 (0.4–4.6)	1.2 (0.6–2.5)
6198: Occupations in labouring and other elemental work, services	1871/1931	66/63	0.9 (0.6–1.3)	0.9 (0.6–1.3)	60/47	1.0 (0.7–1.6)	1.0 (0.7–1.6)	6/16	0.3 (0.1–0.9)^b^	0.6 (0.3–1.2)
71: Farming. Horticultural and animal-husbandry occupations	1840/1860	97/134	0.7 (0.5–1.0)^b^	0.8 (0.6–1.0)	59/82	0.7 (0.5–1.1)	0.8 (0.5–1.1)	38/52	0.7 (0.4–1.1)	0.8 (0.5–1.2)
711: Farmers	1903/1941	34/53	0.6 (0.4–1.0)^b^	0.7 (0.4–1.0)	14/31	0.4 (0.2–0.8)^b^	0.5 (0.3–0.9)^b^	20/22	0.9 (0.5–1.9)	1.0 (0.5–1.7)
7111: General farmers	1923/1974	14/20	0.6 (0.3–1.2)	0.7 (0.4–1.3)	5/12	0.3 (0.1–0.9)^b^	0.6 (0.3–1.2)	9/8	1.1 (0.4–3.2)	1.0 (0.5–2.1)
7111110: Farmer, general (agric.)	1923/1974	14/20	0.6 (0.3–1.2)	0.7 (0.4–1.3)	5/12	0.3 (0.1–0.9)^b^	0.6 (0.3–1.2)	9/8	1.1 (0.4–3.2)	1.0 (0.5–2.1)
7183: Field crop and vegetable-growing workers	1932/1978	5/16	0.3 (0.1–0.9)^b^	0.6 (0.3–1.2)	4/11	0.4 (0.1–1.4)	0.7 (0.3–1.6)	1/5	0.2 (0.0–1.6)	0.8 (0.3–1.9)
7183122: Farm worker, vegetable (agric.)	1935/1983	2/11	0.2 (0.0–0.9)^b^	0.6 (0.3–1.5)	2/7	0.4 (0.1–2.4)	0.8 (0.3–1.9)	0/4		
75: Forestry and logging occupations	1874/1964	63/30	1.9 (1.2–3.0)^b^	1.7 (1.1–2.6)^b^	46/23	1.7 (1.0–3.0)	1.5 (0.9–2.5)	17/7	2.4 (0.9–6.2)	1.6 (0.8–3.1)
751: Forestry and logging occupations	1874/1964	63/30	1.9 (1.2–3.0)^b^	1.7 (1.1–2.6)^b^	46/23	1.7 (1.0–3.0)	1.5 (0.9–2.5)	17/7	2.4 (0.9–6.2)	1.6 (0.8–3.1)
7513: Timber cutting and related occupations	1884/1969	53/25	1.9 (1.1–3.2)^b^	1.6 (1.0–2.6)^b^	38/20	1.7 (0.9–3.0)	1.5 (0.9–2.4)	15/5	2.8 (0.9–8.4)	1.6 (0.8–3.3)
7513122: Logger, all-round (forest. & log.)	1884/1970	53/24	2.0 (1.2–3.4)^b^	1.7 (1.1–2.7)^b^	38/19	1.8 (1.0–3.2)	1.5 (0.9–2.5)	15/5	2.8 (0.9–8.4)	1.6 (0.8–3.3)
8161: Mixing and blending occupations, chemicals and related materials	1919/1986	18/8	3.6 (1.2–10.8)^b^	1.8 (0.9–3.7)	12/4	8.0 (1.3–50.2)^b^	1.6 (0.7–3.8)	6/4	1.9 (0.4–7.7)	1.2 (0.6–2.8)
8161218: Mixer (chem., n.e.c.; paint & varn.)	1926/1991	11/3	8.0 (1.2–53.8)^b^	1.6 (0.6–3.7)	8/2	6.2 (0.8–47.8)	1.4 (0.6–3.5)	3/1	20.6 (0.22.7)	
8529: Other fabricating and assembling occupations, metal products, n.e.c.	1922/1986	15/8	1.8 (0.6–4.9)	1.3 (0.6–2.7)	8/7	0.8 (0.2–2.5)	0.9 (0.4–1.9)	7/1	19.8 (1.0.5)^b^	1.4 (0.5–3.4)
858: Mechanics and repairmen, n.e.c.	1782/1811	155/183	0.8 (0.6–1.1)	0.8 (0.6–1.1)	63/64	1.1 (0.7–1.6)	1.1 (0.7–1.6)	92/119	0.7 (0.5–1.0)^b^	0.7 (0.5–1.0)^b^
8581118: Industrial-truck mechanic (mech. Equip., n.e.c.)	1934/1983	3/11	0.2 (0.0–0.8)^b^	0.6 (0.3–1.3)	1/4	0.4 (0.0–4.2)	0.9 (0.4–2.2)	2/7	0.1 (0.0–0.8)^b^	0.6 (0.3–1.4)
8582: Aircraft mechanics and repairmen	1936/1976	1/18	0.1 (0.0–0.7)^b^	0.6 (0.3–1.6)	0/6			1/12	0.1 (0.0–1.1)	0.7 (0.3–1.7)
8595: Painting and decorating occupations, except construction	1902/1975	35/19	1.9 (1.0–3.5)	1.6 (0.9–2.6)	27/9	3.0 (1.3–7.0)^b^	1.9 (1.0–3.6)	8/10	0.8 (0.3–2.4)	0.9 (0.4–1.9)
873: Electrical power, lighting and wire communications equipment erecting, installing and repairing occupations	1852/1924	85/70	1.3 (0.9–1.8)	1.2 (0.9–1.8)	41/28	2.0 (1.1–3.6)^b^	1.7 (1.0–2.8)^b^	44/42	0.9 (0.6–1.5)	1.0 (0.6–1.5)
8739: Electrical power, lighting and wire communications equipment erecting, installing and repairing occupations, n.e.c.	1933/1980	4/14	0.3 (0.1–0.8)^b^	0.6 (0.3–1.2)	2/8	0.2 (0.0–1.1)	0.7 (0.3–1.6)	2/6	0.3 (0.1–1.6)	0.7 (0.3–1.7)
8784: Plasterers and related occupations	1933/1985	4/9	0.2 (0.1–0.9)^b^	0.6 (0.3–1.3)	0/5			4/4	0.4 (0.1–1.8)	0.8 (0.3–1.7)
8791: Pipefitting, plumbing and related occupations, n.e.c.	1886/1954	51/40	1.7 (1.0–2.8)^b^	1.5 (1.0–2.4)	28/19	1.8 (0.9–3.5)	1.5 (0.8–2.6)	23/21	1.6 (0.7–3.4)	1.3 (0.7–2.4)
8799: Other construction trades occupations, n.e.c.	1877/1948	60/46	1.8 (1.1–2.9)^b^	1.6 (1.0–2.5)^b^	36/26	1.6 (0.9–3.0)	1.4 (0.8–2.4)	24/20	2.1 (0.9–4.6)	1.6 (0.8–2.9)
911: Air transport operating operations	1922/1974	15/20	0.6 (0.3–1.3)	0.7 (0.4–1.3)	11/9	1.3 (0.5–3.6)	1.2 (0.6–2.3)	4/11	0.2 (0.1–0.7)^b^	0.6 (0.3–1.2)
915: Water transport operating occupations	1923/1971	14/23	0.6 (0.3–1.2)	0.7 (0.4–1.3)	7/18	0.3 (0.1–0.8)^b^	0.6 (0.3–1.1)	7/5	1.7 (0.5–6.3)	1.2 (0.6–2.7)
9173: Taxi drivers and chauffeurs	1870/1937	67/57	1.2 (0.8–1.8)	1.2 (0.8–1.7)	24/29	0.8 (0.4–1.4)	0.8 (0.5–1.4)	43/28	1.8 (1.0–3.2)^b^	1.6 (0.9–2.6)
9175129: Solid waste collection truck driver (motor trans.)	1935/1985	2/9	0.2 (0.0–0.9)^b^	0.6 (0.3–1.5)	2/7	0.2 (0.0–1.2)	0.7 (0.3–1.6)	0/2		
9179118: Dispatcher, motor vehicles (motor trans.)	1925/1991	12/3	8.4 (1.2–57.6)^b^	1.6 (0.6–3.8)	9/2	6.8 (0.9–53.9)	1.4 (0.6–3.5)	3/1	18.6 (0.22.5)	
9918: Occupations in labouring and other elemental work, n.e.c.	1920/1982	17/12	2.7 (0.9–7.9)	1.6 (0.8–3.3)	12/7	4.1 (1.0–16.6)^b^	1.6 (0.7–3.6)	5/5	1.3 (0.2–6.7)	1.1 (0.5–2.5)
9918110: Labourer, municipal (gov. Serv.)	1920/1983	17/11	3.4 (1.0–10.8)^b^	1.7 (0.8–3.5)	12/6	6.5 (1.2–33.5)^b^	1.6 (0.7–3.8)	5/5	1.3 (0.2–6.7)	1.1 (0.5–2.5)

^a^Occupations selected had at least one significantly elevated or reduced association with prostate cancer among duration of employment categories (ever, <10 years or ≥10 years). Odds ratios adjusted for age, first-degree family history of prostate cancer, ancestry, screening for prostate cancer, annual household income, highest level of education attained, level of physical activity, alcohol intake and body mass index

^b^The 95 % confidence interval excludes the null value. n.e.c.: not elsewhere classified

**Table 3:** Associations between selected occupations and risk of high grade prostate cancer^a^

CCDO code and description	Never	Ever employed			<10 years employed			≥10 years employed		
	Ca/Co	Ca/Co	OR (95 % CI)	OR SB (95 % CI)	Ca/Co	OR (95 % CI)	OR SB (95 % CI)	Ca/Co	OR (95 % CI)	OR SB (95 % CI)
1135122: Credit manager (prof. & tech., n.e.c.)	520/1990	4/4	4.0 (1.0–17.0)	1.6 (0.7–3.7)	2/1	12.6 (1.1.4)^b^		2/3	2.2 (0.3–13.8)	1.3 (0.6–3.1)
1137: Sales and advertising management occupations	505/1931	19/63	1.3 (0.7–2.2)	1.2 (0.8–2.0)	12/22	2.1 (1.0–4.5)^b^	1.7 (0.9–3.1)	7/41	0.7 (0.3–1.7)	0.9 (0.5–1.7)
1137118: Manager, sales (prof. & tech., n.e.c.)	508/1945	16/49	1.4 (0.8–2.6)	1.3 (0.8–2.2)	10/17	2.8 (1.2–6.5)^b^	1.9 (1.0–3.6)^b^	6/32	0.7 (0.3–1.8)	0.9 (0.5–1.8)
2163: Draughtsmen	512/1949	12/45	1.2 (0.6–2.4)	1.2 (0.7–2.0)	8/15	2.6 (1.0–6.6)^b^	1.8 (0.9–3.4)	4/30	0.6 (0.2–1.7)	0.8 (0.4–1.7)
23: Occupations in social sciences and related fields	494/1914	30/80	1.8 (1.1–3.0)^b^	1.7 (1.1–2.6)^b^	17/30	2.4 (1.3–4.6)^b^	1.9 (1.1–3.3)^b^	13/50	1.4 (0.7–2.7)	1.3 (0.8–2.3)
2349: Occupations in law and jurisprudence, n.e.c.	520/1990	4/4	4.3 (0.9–19.9)	1.6 (0.7–3.7)	4/1	12.2 (1.3.2)^b^	1.7 (0.7–4.1)	0/3		
2711199: Other university teachers	520/1992	4/2	7.0 (1.2–39.6)^b^	1.7 (0.7–4.0)	3/2	4.8 (0.8–29.4)	1.6 (0.7–3.7)	1/0		
279: Other teaching and related occupations	501/1933	23/61	1.7 (1.0–2.8)	1.5 (1.0–2.4)	10/38	1.2 (0.6–2.6)	1.2 (0.7–2.2)	13/23	2.3 (1.1–4.8)^b^	1.8 (1.0–3.2)
4131134: Accounting clerk (clerical)	514/1976	10/18	2.8 (1.2–6.8)^b^	1.8 (1.0–3.5)	7/14	2.4 (0.9–6.5)	1.6 (0.8–3.3)	3/4	4.8 (0.8–28.6)	1.6 (0.7–3.7)
4131142: Bookkeeping clerk (clerical)	513/1973	11/21	2.8 (1.2–6.3)^b^	1.9 (1.0–3.6)^b^	9/15	3.3 (1.3–8.5)^b^	2.0 (1.0–3.9)^b^	2/6	1.6 (0.3–9.1)	1.2 (0.5–2.9)
4133110: Teller (bank. & finance)	512/1969	12/25	2.1 (1.0–4.4)	1.6 (0.9–3.0)	12/24	2.1 (1.0–4.4)^b^	1.7 (0.9–3.0)	0/1		
5133114: Pharmaceutical representative (whole. Trade)	520/1986	4/8	1.9 (0.6–6.5)	1.3 (0.6–2.9)	4/4	4.6 (1.1–19.3)^b^	1.8 (0.8–4.0)	0/4		
5135178: Salesperson, wearing apparel (ret. Trade; whole. Trade)	514/1980	10/14	3.2 (1.3–7.6)^b^	2.0 (1.0–3.8)^b^	7/10	3.0 (1.1–8.3)^b^	1.8 (0.9–3.6)	3/4	3.8 (0.7–19.4)	1.6 (0.7–3.6)
5145: Service station attendants	511/1982	13/12	4.3 (1.8–10.4)^b^	2.4 (1.2–4.5)^b^	11/11	4.0 (1.6–10.2)^b^	2.2 (1.1–4.3)^b^	2/1	7.0 (0.6–78.7)	
5145110: Service-station attendant (motor vehicle; ret. Trade)	511/1982	13/12	4.4 (1.8–10.5)^b^	2.4 (1.2–4.5)^b^	11/11	4.0 (1.6–10.3)^b^	2.2 (1.1–4.3)^b^	2/1	7.5 (0.7–84.2)	
5172: Real estate salesmen	508/1960	16/34	1.8 (0.9–3.3)	1.5 (0.9–2.6)	10/16	2.4 (1.0–5.5)^b^	1.8 (0.9–3.3)	6/18	1.2 (0.5–3.3)	1.2 (0.6–2.4)
5172118: Salesperson, real estate (insur. & real estate)	508/1965	16/29	2.0 (1.1–3.9)^b^	1.7 (1.0–2.9)	10/14	2.9 (1.2–6.8)^b^	1.9 (1.0–3.7)	6/15	1.4 (0.5–3.7)	1.2 (0.6–2.5)
6112: Police officers and detectives, government	513/1964	11/30	1.4 (0.7–2.9)	1.3 (0.7–2.3)	5/5	4.0 (1.0–15.1)^b^	1.8 (0.8–3.9)	6/25	0.9 (0.4–2.3)	1.0 (0.5–2.0)
75: Forestry and logging occupations	500/1964	24/30	2.5 (1.4–4.5)^b^	2.0 (1.2–3.3)^b^	15/23	2.0 (1.0–4.0)	1.6 (0.9–2.9)	9/7	4.4 (1.5–12.7)^b^	2.1 (1.0–4.3)^b^
751: Forestry and logging occupations	500/1964	24/30	2.5 (1.4–4.5)^b^	2.0 (1.2–3.3)^b^	15/23	2.0 (1.0–4.0)	1.6 (0.9–2.9)	9/7	4.4 (1.5–12.7)^b^	2.1 (1.0–4.3)^b^
7513: Timber cutting and related occupations	504/1969	20/25	2.5 (1.3–4.7)^b^	1.9 (1.1–3.3)^b^	12/20	1.8 (0.9–4.0)	1.5 (0.8–2.8)	8/5	5.2 (1.5–17.6)^b^	2.1 (1.0–4.4)
7513122: Logger, all-round (forest. & log.)	504/1970	20/24	2.6 (1.4–5.0)^b^	2.0 (1.2–3.4)^b^	12/19	2.0 (0.9–4.2)	1.6 (0.9–2.9)	8/5	5.2 (1.5–17.6)^b^	2.1 (1.0–4.4)
8131: Metal smelting, converting and refining furnacemen	522/1991	2/3	2.3 (0.3–16.8)	1.3 (0.5–3.0)	1/2	1.1 (0.1–12.7)		1/1	53.7 (13.2.3)^b^	
821/822: Food, beverage and related processing occupations	503/1882	21/112	0.5 (0.3–0.9)^b^	0.6 (0.4–1.0)^b^	12/74	0.5 (0.2–0.9)^b^	0.6 (0.4–1.1)	9/38	0.6 (0.3–1.4)	0.8 (0.4–1.5)
825: Pulp and papermaking and related occupations	520/1991	4/3	6.3 (1.1–35.6)^b^	1.7 (0.7–3.9)	3/2	4.2 (0.7–26.3)	1.5 (0.6–3.6)	1/1	56.4 (0.277.5)	
826/827: Textile processing occupations	499/1948	25/46	1.8 (1.0–3.2)^b^	1.6 (1.0–2.6)	15/29	1.5 (0.8–2.9)	1.4 (0.8–2.4)	10/17	2.8 (1.1–6.8)^b^	1.8 (0.9–3.6)
8278: Occupations in labouring and other elemental work, textile processing	519/1991	5/3	7.8 (1.5–41.2)^b^	1.8 (0.8–4.2)	4/3	6.2 (1.1–35.2)^b^	1.7 (0.7–4.0)	1/0		
8551: Patternmaking, marking and cutting occupations	507/1959	17/35	1.6 (0.9–3.0)	1.4 (0.8–2.4)	6/24	0.9 (0.3–2.2)	1.0 (0.5–1.9)	11/11	3.3 (1.3–8.6)^b^	2.0 (1.0–3.9)^b^
8595: Painting and decorating occupations, except construction	514/1975	10/19	1.7 (0.8–3.9)	1.4 (0.8–2.7)	8/9	2.9 (1.0–8.1)^b^	1.8 (0.9–3.6)	2/10	0.6 (0.1–3.2)	1.0 (0.4–2.2)
873: Electrical power, lighting and wire communications equipment erecting, installing and repairing occupations	498/1924	26/70	1.4 (0.9–2.3)	1.4 (0.9–2.1)	12/28	2.2 (1.0–4.7)^b^	1.7 (0.9–3.1)	14/42	1.1 (0.6–2.1)	1.1 (0.6–1.9)
8799: Other construction trades occupations, n.e.c.	505/1948	19/46	2.0 (1.0–3.6)^b^	1.7 (1.0–2.8)	12/26	1.9 (0.9–4.1)	1.6 (0.8–2.9)	7/20	2.0 (0.7–5.6)	1.5 (0.7–3.0)
9171: Bus drivers	507/1954	17/40	1.6 (0.9–3.0)	1.5 (0.9–2.5)	9/14	2.9 (1.1–7.3)^b^	1.9 (0.9–3.6)	8/26	1.1 (0.5–2.5)	1.1 (0.6–2.1)
9171110: Bus driver (motor trans.)	507/1954	17/40	1.6 (0.9–3.0)	1.5 (0.9–2.5)	9/14	2.9 (1.1–7.3)^b^	1.9 (0.9–3.6)	8/26	1.1 (0.5–2.5)	1.1 (0.6–2.1)
9179118: Dispatcher, motor vehicles (motor trans.)	519/1991	5/3	12.7 (1.6.5)^b^	1.7 (0.7–4.2)	5/2	13.1 (1.5.1)^b^	1.7 (0.7–4.2)	0/1		
9310: Foremen/women, material handling and related occupations, n.e.c.	519/1989	5/5	5.1 (1.2–21.5)^b^	1.8 (0.8–4.0)	2/3	3.6 (0.5–28.4)	1.4 (0.6–3.4)	3/2	8.5 (0.9–81.2)	1.6 (0.6–3.8)
955: Electronic and related communications equipment operating occupations, n.e.c.	515/1985	9/9	3.5 (1.4–9.2)^b^	2.0 (1.0–4.0)^b^	7/6	3.8 (1.2–11.6)^b^	1.9 (0.9–4.0)	2/3	2.8 (0.5–17.4)	1.4 (0.6–3.3)

^a^High grade defined by Gleason score ≥8 or 4 + 3. Occupations selected had at least one significantly elevated or reduced association with prostate cancer among duration of employment categories (ever, <10 years or ≥10 years). Odds ratios estimated using multinomial models adjusted for age, first–degree family history of prostate cancer, ancestry, screening for prostate cancer, annual household income, highest level of education attained, level of physical activity, alcohol intake (drink–years) and body mass index

^b^The 95 % confidence interval excludes the null value. n.e.c.: not elsewhere classified

Two 2-digit categories were associated with statistically elevated ORs for ever employment in the following groups: Occupations in social sciences (OR 1.6, 95 % CI: 1.1–2.2) and Forestry and logging occupations (OR 1.9, 95 % CI: 1.2–3.0). Subgroups with increased PCa risk within these categories include educational counsellors (OR 3.0, 95 % CI 1.1–8.3) and loggers (OR 2.0, 95 % CI 1.2–3.4). Elevated ORs were also found for ever employment as Police officers and detectives (OR 1.8, 95 % CI 1.1–2.9), Mixing and blending occupations (OR 3.6, 95 % CI 1.2–10.8), Governmental inspectors and regulatory officers (OR 2.7, 95 % CI 1.2–6.2), employment <10 years in Painting and decorating occupations excepting construction (OR 3.0, 95 % CI 1.3–7.0), and in occupations associated with administration and sales, such as Receptionists, General office clerks for ever and <10 years employed, and Commodities sales clerks employed <10 years.

Associations for high grade PCa, estimated using multinomial models, were observed for ever employment in Social sciences occupations (OR 1.8, 95 % CI 1.1–3.0) and Forestry and logging (OR 2.5, 95 % CI 1.4–4.5). For the latter, the association was the strongest for employment ≥10 years (OR 4.4, 95 % CI 1.5–12.7). Other groups where associations were mainly limited to high grade PCa were Service station attendants (OR ever 4.3, 95 % CI 1.8–10.4), Textile processing occupations (OR ever 1.8, 95 % CI 1.1–3.2), and Bus drivers (OR <10 years 2.9, 95 % CI 1.1–7.3).

Regarding decreased PCa risk, negative associations were found for employment in Farming; specific occupations within farming where such associations were observed were General farmers (OR <10 years 0.3, 95 % CI 0.1–0.9), and Field crop and Vegetable growing workers (OR ever 0.3, 95 % CI 0.1–0.9). Decreased PCa risk was also found in occupations associated with Aircraft and Air transportation, and for ever and <10 years employment in Electrical engineers.

Applying SB adjustment for the analysis by occupation led to an attenuation of risk estimates, especially in occupations with few subjects ever employed. For example, the number of categories with a statistically significant association between ever employment and PCa risk saw a threefold reduction, from 37 to 12. The more robust associations for total PCa risk were found for ever employment in Occupations in social sciences (ORsb 1.5, 95 % CI 1.1–2.1), Police officers (ORsb 1.7, 95 % CI 1.0–2.7) and Forestry and logging occupations (ORsb 1.7, 95 % CI 1.1–2.6). For high grade PCa, these included Occupations in social sciences, Forestry and logging, Bookkeeping clerks and Service station attendants. Decreased PCa risk associated with employment <10 years as Farmer also remained robust to shrinkage (ORsb 0.5, 95 % CI 0.3–0.9).

For the analysis by industry title, the number of 2, 3 and 4-digit industry categories with at least one subject ever employed totaled 1125, with 613 (54 %) featuring at least 10 subjects ever employed. Associations between PCa risk and employment in these 613 categories are presented in [Additional file 3: Table S2]; categories with at least one statistically significant association for total PCa are presented in Tables 4 and 5 for high grade PCa. The remaining 512 industry groups excluded from the analysis are listed in [Additional file 2: Table S3].

**Table 4:** Associations between selected industries and risk of prostate cancer (all cancers)^a^

SIC code and description	Never	Ever employed			<10 years employed			≥10 years employed		
	Ca/Co	Ca/Co	OR (95 % CI)	OR SB (95 % CI)	Ca/Co	OR (95 % CI)	OR SB (95 % CI)	Ca/Co	OR (95 % CI)	OR SB (95 % CI)
01: Agricultural industries	1864/1886	73/108	0.6 (0.4–0.9)^b^	0.6 (0.5–0.9)^b^	43/66	0.6 (0.4–0.9)^b^	0.7 (0.4–1.0)^b^	30/42	0.6 (0.4–1.1)	0.7 (0.4–1.1)
0119: Livestock combination farms	1932/1980	5/14	0.3 (0.1–0.9)^b^	0.6 (0.3–1.3)	2/7	0.2 (0.0–1.0)	0.7 (0.3–1.6)	3/7	0.4 (0.1–2.4)	0.8 (0.3–1.9)
04: Logging industry	1881/1962	56/32	1.7 (1.0–2.8)^b^	1.5 (1.0–2.4)	43/23	1.7 (0.9–2.9)	1.5 (0.9–2.4)	13/9	1.8 (0.7–4.7)	1.4 (0.7–2.7)
041: Logging industry	1881/1962	56/32	1.7 (1.0–2.8)^b^	1.5 (1.0–2.4)	43/23	1.7 (0.9–2.9)	1.5 (0.9–2.4)	13/9	1.8 (0.7–4.7)	1.4 (0.7–2.7)
103: Fruit and vegetable industries	1936/1985	1/9	0.1 (0.0–0.5)^b^	0.6 (0.3–1.5)	1/7	0.1 (0.0–0.8)^b^	0.7 (0.3–1.6)	0/2		
1031: Canned and preserved fruit and vegetable industry	1936/1985	1/9	0.1 (0.0–0.5)^b^	0.6 (0.3–1.5)	1/7	0.1 (0.0–0.8)^b^	0.7 (0.3–1.6)	0/2		
2619: Other household furniture industries	1922/1989	15/5	3.9 (1.1–13.4)^b^	1.7 (0.8–3.7)	11/5	3.1 (0.8–11.0)	1.5 (0.7–3.4)	4/0		
27: Paper and allied products industries	1874/1943	63/51	1.3 (0.9–2.0)	1.3 (0.9–1.9)	40/37	1.0 (0.6–1.7)	1.0 (0.6–1.6)	23/14	2.3 (1.1–5.0)^b^	1.7 (0.9–3.1)
2711: Pulp industry	1911/1972	26/22	1.3 (0.7–2.4)	1.2 (0.7–2.0)	16/18	0.8 (0.4–1.7)	0.9 (0.5–1.6)	10/4	5.6 (1.1–27.5)^b^	1.6 (0.7–3.8)
3039: Other ornamental and architectural metal products industries	1927/1991	10/3	5.9 (1.0–33.6)^b^	1.6 (0.7–3.7)	7/2	13.2 (1.3.8)^b^	1.5 (0.6–3.7)	3/1	1.6 (0.2–16.1)	
3199: Other machinery and equipment industries n.e.c.	1920/1966	17/28	0.6 (0.3–1.1)	0.7 (0.4–1.2)	11/22	0.4 (0.2–1.0)^b^	0.6 (0.3–1.1)	6/6	1.0 (0.3–3.6)	1.0 (0.5–2.2)
32: Transportation equipment industries	1809/1831	128/163	0.8 (0.6–1.0)	0.8 (0.6–1.0)	80/90	0.9 (0.6–1.2)	0.9 (0.6–1.2)	48/73	0.7 (0.4–1.0)^b^	0.7 (0.5–1.0)
321: Aircraft and aircraft parts industry	1881/1897	56/97	0.6 (0.4–0.9)^b^	0.6 (0.5–0.9)^b^	32/48	0.7 (0.4–1.2)	0.8 (0.5–1.2)	24/49	0.5 (0.3–0.8)^b^	0.6 (0.4–0.9)^b^
3211: Aircraft and aircraft parts industry	1881/1897	56/97	0.6 (0.4–0.9)^b^	0.6 (0.5–0.9)^b^	32/48	0.7 (0.4–1.2)	0.8 (0.5–1.2)	24/49	0.5 (0.3–0.8)^b^	0.6 (0.4–0.9)^b^
354: Concrete products industries	1925/1987	12/7	4.4 (1.0–18.5)^b^	1.6 (0.7–3.7)	7/6	2.3 (0.5–11.2)	1.3 (0.6–3.0)	5/1	28.1 (0.89.3)	1.3 (0.5–3.3)
356: Glass and glass products industries	1925/1976	12/18	0.6 (0.3–1.4)	0.8 (0.4–1.4)	9/9	1.2 (0.4–3.4)	1.1 (0.5–2.2)	3/9	0.2 (0.1–1.0)^b^	0.6 (0.3–1.4)
3561: Primary glass and glass containers industry	1929/1977	8/17	0.5 (0.2–1.2)	0.7 (0.3–1.3)	6/9	0.9 (0.3–2.8)	1.0 (0.4–2.0)	2/8	0.2 (0.0–0.9)^b^	0.6 (0.3–1.5)
424: Plumbing, heating and air conditioning, mechanical work	1887/1957	50/37	1.7 (1.0–2.8)^b^	1.5 (1.0–2.4)	23/16	1.7 (0.8–3.6)	1.4 (0.8–2.6)	27/21	1.7 (0.8–3.3)	1.4 (0.8–2.5)
427: Interior and finishing work	1891/1958	46/36	1.7 (1.0–2.9)^b^	1.5 (1.0–2.4)	17/13	1.8 (0.8–4.4)	1.4 (0.7–2.7)	29/23	1.6 (0.9–3.2)	1.4 (0.8–2.4)
451: Air transport industries	1923/1957	14/37	0.4 (0.2–0.9)^b^	0.6 (0.3–1.0)	4/10	0.5 (0.1–1.9)	0.8 (0.4–1.8)	10/27	0.4 (0.2–0.9)^b^	0.6 (0.3–1.1)
4511: Scheduled air transport industry	1923/1959	14/35	0.5 (0.2–0.9)^b^	0.6 (0.3–1.0)	4/9	0.6 (0.1–2.4)	0.9 (0.4–1.9)	10/26	0.4 (0.2–0.9)^b^	0.6 (0.3–1.1)
454: Water transport industries	1925/1964	12/30	0.4 (0.2–0.8)^b^	0.6 (0.3–1.0)	6/21	0.3 (0.1–0.8)^b^	0.6 (0.3–1.1)	6/9	0.6 (0.2–2.0)	0.8 (0.4–1.8)
4541: Freight and passenger water transport industry	1925/1966	12/28	0.4 (0.2–0.9)^b^	0.6 (0.3–1.0)	6/19	0.3 (0.1–0.8)^b^	0.6 (0.3–1.1)	6/9	0.6 (0.2–2.0)	0.8 (0.4–1.8)
4571: Urban transit systems industry	1888/1949	49/45	1.1 (0.7–1.8)	1.1 (0.7–1.6)	21/11	2.4 (1.0–5.8)^b^	1.7 (0.9–3.2)	28/34	0.8 (0.5–1.4)	0.8 (0.5–1.4)
5512: Trucks and buses, wholesale	1932/1986	5/8	0.5 (0.1–1.8)	0.8 (0.4–1.7)	1/7	0.1 (0.0–1.0)^b^	0.7 (0.3–1.8)	4/1	2.5 (0.3–23.3)	1.2 (0.5–2.9)
561: Metal and metal products, wholesale	1933/1981	4/13	0.3 (0.1–1.1)	0.6 (0.3–1.4)	1/9	0.1 (0.0–0.8)^b^	0.7 (0.3–1.7)	3/4	0.9 (0.2–4.5)	1.0 (0.4–2.3)
599: Other products n.e.c., wholesale	1919/1985	18/9	2.5 (1.0–6.0)	1.6 (0.8–3.2)	11/4	4.9 (1.1–21.6)^b^	1.6 (0.7–3.7)	7/5	1.4 (0.4–4.6)	1.1 (0.5–2.5)
5999: Other products n.e.c., wholesale	1922/1988	15/6	3.4 (1.1–10.4)^b^	1.7 (0.8–3.6)	8/3	6.5 (0.9–46.1)	1.5 (0.6–3.6)	7/3	2.2 (0.5–8.9)	1.3 (0.6–2.9)
611: Shoe stores	1931/1980	6/14	0.3 (0.1–1.0)^b^	0.6 (0.3–1.3)	4/8	0.3 (0.1–1.2)	0.7 (0.3–1.5)	2/6	0.4 (0.1–2.1)	0.8 (0.3–1.9)
6111: Shoe stores	1931/1980	6/14	0.3 (0.1–1.0)^b^	0.6 (0.3–1.3)	4/8	0.3 (0.1–1.2)	0.7 (0.3–1.5)	2/6	0.4 (0.1–2.1)	0.8 (0.3–1.9)
70: Deposit accepting intermediary industries	1834/1912	103/82	1.3 (0.9–1.9)	1.3 (0.9–1.8)	54/41	1.7 (1.0–2.7)^b^	1.5 (1.0–2.4)	49/41	1.1 (0.7–1.7)	1.1 (0.7–1.6)
702: Chartered banks and other banking-type intermediaries	1840/1919	97/75	1.4 (1.0–2.0)	1.4 (1.0–1.9)	53/39	1.8 (1.1–2.9)^b^	1.6 (1.0–2.4)^b^	44/36	1.1 (0.7–1.8)	1.1 (0.7–1.7)
7021: Chartered banks	1843/1921	94/73	1.4 (1.0–2.0)	1.3 (1.0–1.9)	53/38	1.8 (1.1–2.9)^b^	1.6 (1.0–2.5)^b^	41/35	1.1 (0.7–1.8)	1.1 (0.7–1.6)
72: Investment intermediary industries	1916/1977	21/17	1.9 (0.9–4.1)	1.5 (0.8–2.7)	14/7	3.7 (1.1–12.2)^b^	1.7 (0.8–3.6)	7/10	1.0 (0.3–2.9)	1.0 (0.5–2.1)
8153: Taxation administration, federal	1922/1986	15/8	3.2 (1.0–9.5)^b^	1.7 (0.8–3.5)	7/4	4.9 (0.7–33.8)	1.4 (0.6–3.4)	8/4	2.4 (0.6–9.4)	1.4 (0.6–3.1)
82: Provincial and territorial government service industries	1781/1892	156/102	1.5 (1.1–2.0)^b^	1.5 (1.1–2.0)^b^	72/54	1.3 (0.9–2.0)	1.3 (0.9–1.8)	84/48	1.8 (1.2–2.7)^b^	1.6 (1.1–2.4)^b^
822: Protective services (provincial)	1893/1971	44/23	1.8 (1.0–3.2)^b^	1.6 (1.0–2.5)	23/14	1.6 (0.7–3.4)	1.4 (0.7–2.5)	21/9	2.0 (0.9–4.7)	1.5 (0.8–2.9)
8225: Regulatory services, provincial	1920/1989	17/5	6.1 (1.5–24.8)^b^	1.8 (0.8–4.1)	8/3	17.0 (1.2.8)^b^	1.4 (0.6–3.6)	9/2	3.7 (0.8–17.8)	1.5 (0.6–3.4)
825: General administrative services (provincial)	1891/1966	46/28	1.7 (1.0–2.8)	1.5 (0.9–2.4)	21/16	1.0 (0.5–2.1)	1.0 (0.6–1.8)	25/12	3.0 (1.3–7.0)^b^	1.9 (1.0–3.6)
92: Food and beverage service industries	1786/1796	151/198	0.7 (0.6–0.9)^b^	0.8 (0.6–1.0)^b^	91/115	0.7 (0.5–1.0)	0.8 (0.6–1.0)	60/83	0.8 (0.5–1.1)	0.8 (0.6–1.1)
921: Food services	1818/1832	119/162	0.7 (0.6–1.0)^b^	0.8 (0.6–1.0)^b^	75/99	0.7 (0.5–1.0)	0.7 (0.5–1.0)	44/63	0.8 (0.5–1.2)	0.8 (0.5–1.2)
9212: Restaurants, unlicensed (including drive-ins)	1906/1931	31/63	0.5 (0.3–0.8)^b^	0.6 (0.4–0.9)^b^	24/41	0.6 (0.3–1.0)	0.7 (0.4–1.1)	7/22	0.3 (0.1–0.9)^b^	0.6 (0.3–1.2)
984: Labour organizations	1921/1974	16/20	0.7 (0.3–1.4)	0.8 (0.4–1.4)	5/13	0.3 (0.1–0.9)^b^	0.6 (0.3–1.2)	11/7	1.3 (0.5–3.7)	1.2 (0.6–2.4)
9841: Labour organizations	1921/1974	16/20	0.7 (0.3–1.4)	0.8 (0.4–1.4)	5/13	0.3 (0.1–0.9)^b^	0.6 (0.3–1.2)	11/7	1.3 (0.5–3.7)	1.2 (0.6–2.4)
986: Civic and fraternal organizations	1928/1972	9/22	0.4 (0.2-0.8)^b^	0.6 (0.3–1.1)	7/13	0.4 (0.2–1.2)	0.7 (0.3–1.4)	2/9	0.2 (0.0–1.2)	0.7 (0.3–1.6)
9861: Civic and fraternal organizations	1928/1972	9/22	0.4 (0.2–0.8)^b^	0.6 (0.3–1.1)	7/13	0.4 (0.2–1.2)	0.7 (0.3–1.4)	2/9	0.2 (0.0–1.2)	0.7 (0.3–1.6)

^**a**^Industries selected had at least one significantly elevated or reduced association with prostate cancer among duration of employment categories (ever, <10 years or ≥10 years). Odds ratios adjusted for age, first–degree family history of prostate cancer, ancestry, screening for prostate cancer, annual household income, highest level of education attained, level of physical activity, alcohol intake and body mass index

^b^The 95 % confidence interval excludes the null value. n.e.c.: not elsewhere classified

**Table 5:** Associations between selected industries and risk of high grade prostate cancer^a^

SIC code and description	Never	Ever employed			<10 years employed			≥10 years employed		
	Ca/Co	Ca/Co	OR (95 % CI)	OR SB (95 % CI)	Ca/Co	OR (95 % CI)	OR SB (95 % CI)	Ca/Co	OR (95 % CI)	OR SB (95 % CI)
0111: Dairy farms	518/1988	6/6	3.5 (0.9–12.8)	1.7 (0.8–3.7)	6/4	4.5 (1.1–18.9)^b^	1.8 (0.8–4.0)	0/2		
016: Horticultural specialties	520/1988	4/6	2.7 (0.7–11.3)	1.5 (0.7–3.4)	4/2	9.8 (1.1–87.4)^b^	1.6 (0.7–4.0)	0/4		
04: Logging industry	503/1962	21/32	2.1 (1.1–3.9)^b^	1.8 (1.1–3.0)^b^	14/23	1.8 (0.9–3.7)	1.5 (0.9–2.8)	7/9	3.1 (1.0–9.3)^b^	1.8 (0.9–3.7)
041: Logging industry	503/1962	21/32	2.1 (1.1–3.9)^b^	1.8 (1.1–3.0)^b^	14/23	1.8 (0.9–3.7)	1.5 (0.9–2.8)	7/9	3.1 (1.0–9.3)^b^	1.8 (0.9–3.7)
0412: Contract logging industry	518/1986	6/8	3.7 (1.1–12.5)^b^	1.8 (0.8–3.9)	4/6	4.2 (0.9–19.0)	1.7 (0.8–3.8)	2/2	2.7 (0.3–21.3)	
104: Dairy products industries	512/1980	12/14	2.5 (1.0–6.0)^b^	1.7 (0.9–3.3)	7/11	1.7 (0.6–5.2)	1.4 (0.6–2.8)	5/3	5.3 (1.0–28.0)^b^	1.7 (0.7–4.0)
25: Wood industries	504/1955	20/39	1.9 (1.0–3.6)^b^	1.7 (1.0–2.8)	17/31	2.2 (1.1–4.4)^b^	1.8 (1.0–3.1)^b^	3/8	1.0 (0.2–4.3)	1.1 (0.5–2.5)
2619: Other household furniture industries	518/1989	6/5	5.5 (1.4–21.9)^b^	1.9 (0.9–4.3)	3/5	3.0 (0.6–14.7)	1.5 (0.6–3.4)	3/0		
291: Primary steel industries	511/1969	13/25	2.1 (1.0–4.5)^b^	1.7 (0.9–3.0)	6/17	1.5 (0.6–4.2)	1.3 (0.6–2.7)	7/8	3.2 (1.1–9.8)^b^	1.8 (0.9–3.8)
2919: Other primary steel industries	518/1988	6/6	4.5 (1.2–16.3)^b^	1.9 (0.8–4.1)	2/4	3.2 (0.4–23.0)	1.4 (0.6–3.4)	4/2	5.7 (1.0–32.7)	1.7 (0.7–4.0)
3039: Other ornamental and architectural metal products industries	520/1991	4/3	8.3 (1.2–55.9)^b^	1.7 (0.7–4.1)	4/2	27.1 (2.3.0)^b^	1.8 (0.7–4.4)	0/1		
3331: Lighting fixture industry	519/1989	5/5	5.2 (1.2–21.9)^b^	1.8 (0.8–4.1)	3/4	4.8 (0.8–29.3)	1.6 (0.7–3.8)	2/1	5.8 (0.5–66.3)	
4123: Hydroelectric power plants and related structures(except transmission lines)	518/1987	6/7	4.9 (1.3–17.7)^b^	1.9 (0.9–4.2)	5/6	4.3 (1.1–16.4)^b^	1.8 (0.8–4.0)	1/1	26.7 (0.19.5)	
4214: Excavating and grading	516/1980	8/14	2.1 (0.8–5.3)	1.5 (0.8–3.0)	5/5	5.9 (1.2–29.5)^b^	1.8 (0.8–4.1)	3/9	1.0 (0.3–4.0)	1.1 (0.5–2.4)
4241: Plumbing	512/1966	12/28	1.8 (0.9–3.9)	1.5 (0.8–2.8)	8/11	3.2 (1.1–8.9)^b^	1.9 (0.9–3.8)	4/17	1.0 (0.3–3.2)	1.1 (0.5–2.3)
456: Truck transport industries	492/1917	32/77	1.5 (1.0–2.4)	1.4 (0.9–2.2)	7/35	0.8 (0.4–2.0)	1.0 (0.5–1.8)	25/42	2.0 (1.1–3.5)^b^	1.7 (1.1–2.8)^b^
4571: Urban transit systems industry	509/1949	15/45	1.2 (0.7–2.3)	1.2 (0.7–2.0)	8/11	3.5 (1.2–9.8)^b^	1.9 (0.9–4.0)	7/34	0.7 (0.3–1.7)	0.9 (0.5–1.7)
492: Gas distribution systems industry	519/1988	5/6	3.7 (1.0–13.8)^b^	1.7 (0.8–3.8)	1/2	2.0 (0.2–26.0)		4/4	4.6 (1.0–21.3)	1.7 (0.8–3.9)
4921: Gas distribution systems industry	519/1988	5/6	3.7 (1.0–13.8)^b^	1.7 (0.8–3.8)	1/2	2.0 (0.2–26.0)		4/4	4.6 (1.0–21.3)	1.7 (0.8–3.9)
599: Other products n.e.c., wholesale	518/1985	6/9	3.1 (1.0–9.5)^b^	1.7 (0.8–3.6)	2/4	3.6 (0.5–25.6)	1.4 (0.6–3.5)	4/5	2.8 (0.7–10.9)	1.5 (0.7–3.4)
5999: Other products n.e.c., wholesale	518/1988	6/6	5.0 (1.4–18.4)^b^	1.9 (0.9–4.2)	2/3	6.4 (0.6–64.8)	1.5 (0.6–3.7)	4/3	4.3 (0.9–21.0)	1.7 (0.7–3.8)
6212: Household furniture stores (without appliances and furnishings)	516/1986	8/8	3.1 (1.1–8.8)^b^	1.8 (0.9–3.7)	5/5	3.2 (0.8–12.3)	1.6 (0.7–3.6)	3/3	2.9 (0.6–14.7)	1.5 (0.6–3.4)
633: Gasoline service stations	506/1968	18/26	2.8 (1.4–5.5)^b^	2.1 (1.2–3.6)^b^	16/22	2.9 (1.4–6.0)^b^	2.1 (1.2–3.7)^b^	2/4	2.0 (0.3–11.6)	1.3 (0.6–3.1)
6331: Gasoline service stations	506/1968	18/26	2.8 (1.4–5.5)^b^	2.1 (1.2–3.6)^b^	16/22	2.9 (1.4–6.0)^b^	2.1 (1.2–3.7)^b^	2/4	2.0 (0.3–11.6)	1.3 (0.6–3.1)
70: Deposit accepting intermediary industries	497/1912	27/82	1.4 (0.9–2.3)	1.4 (0.9–2.1)	16/41	1.9 (1.0–3.7)^b^	1.6 (1.0–2.8)	11/41	1.1 (0.5–2.1)	1.1 (0.6–1.9)
702: Chartered banks and other banking-type intermediaries	498/1919	26/75	1.5 (0.9–2.5)	1.4 (0.9–2.2)	16/39	2.0 (1.1–3.9)^b^	1.7 (1.0–2.9)	10/36	1.1 (0.5–2.3)	1.1 (0.6–2.0)
7021: Chartered banks	498/1921	26/73	1.6 (1.0–2.6)	1.5 (0.9–2.3)	16/38	2.1 (1.1–4.0)^b^	1.7 (1.0–3.0)^b^	10/35	1.2 (0.6–2.4)	1.1 (0.6–2.1)
72: Investment intermediary industries	517/1977	7/17	2.8 (1.0–7.5)^b^	1.8 (0.9–3.5)	4/7	4.5 (1.0–19.4)^b^	1.7 (0.8–3.9)	3/10	2.0 (0.5–8.0)	1.4 (0.6–3.0)
729: Other investment intermediaries	520/1989	4/5	6.0 (1.3–27.7)^b^	1.8 (0.8–4.2)	2/0			2/5	3.0 (0.5–18.3)	1.4 (0.6–3.4)
7299: Other investment intermediaries n.e.c.	520/1990	4/4	6.1 (1.3–27.9)^b^	1.8 (0.8–4.2)	2/0			2/4	3.0 (0.5–18.3)	1.4 (0.6–3.4)
81: Federal government service industries	469/1734	55/260	0.8 (0.6–1.1)	0.8 (0.6–1.1)	33/183	0.7 (0.4–1.0)^b^	0.7 (0.5–1.0)	22/77	1.2 (0.7–1.9)	1.2 (0.7–1.8)
8152: Finance and economic administration, federal	520/1987	4/7	7.7 (1.5–38.7)^b^	1.9 (0.8–4.4)	1/5	3.1 (0.2–42.6)	1.3 (0.5–3.2)	3/2	14.4 (1.6.0)^b^	1.7 (0.7–4.3)
8164: Recreation and culture administration, federal	521/1993	3/1	122.3 (0.554.8)		3/1	137.2 (63.6.9)^b^		0/0		
82: Provincial and territorial government service industries	482/1892	42/102	1.7 (1.2–2.6)^b^	1.6 (1.1–2.4)^b^	22/54	1.6 (0.9–2.8)	1.5 (0.9–2.4)	20/48	1.8 (1.0–3.2)^b^	1.6 (1.0–2.7)^b^
8259: Other general administrative services, provincial	519/1992	5/2	8.0 (1.5–42.9)^b^	1.8 (0.8–4.3)	3/1	8.9 (0.9–88.6)		2/1	7.5 (0.6–86.4)	
83: Local government service industries	483/1856	41/138	1.2 (0.8–1.7)	1.1 (0.8–1.6)	20/44	1.9 (1.1–3.4)^b^	1.7 (1.0–2.8)^b^	21/94	0.8 (0.5–1.4)	0.9 (0.6–1.4)
8362: Social service administration, municipal	519/1988	5/6	4.0 (1.1–15.2)^b^	1.8 (0.8–3.9)	2/3	6.6 (0.7–62.7)	1.5 (0.6–3.7)	3/3	2.9 (0.6–14.8)	1.5 (0.6–3.4)
855: Museums and archives	519/1990	5/4	3.4 (0.8–13.7)	1.6 (0.7–3.6)	5/3	4.9 (1.1–22.4)^b^	1.8 (0.8–4.0)	0/1		
8551: Museums and archives	519/1990	5/4	3.4 (0.8–13.7)	1.6 (0.7–3.6)	5/3	4.9 (1.1–22.4)^b^	1.8 (0.8–4.0)	0/1		
864: Non-institutional social services	516/1975	8/19	1.7 (0.7–4.3)	1.4 (0.7–2.8)	6/11	3.4 (1.1–10.6)^b^	1.8 (0.9–3.8)	2/8	0.6 (0.1–3.0)	1.0 (0.4–2.2)
8642: Child welfare services	519/1988	5/6	2.9 (0.8–10.7)	1.6 (0.7–3.5)	3/1	62.2 (1.83.3)^b^		2/5	1.0 (0.2–5.6)	1.1 (0.5–2.6)
9212: Restaurants, unlicensed (including drive-ins)	516/1931	8/63	0.4 (0.2–1.0)^b^	0.6 (0.3–1.2)	6/41	0.5 (0.2–1.2)	0.7 (0.4–1.4)	2/22	0.3 (0.1–1.4)	0.8 (0.3–1.8)
961: Motion picture, audio and video production and distribution	518/1985	6/9	2.8 (0.9–8.6)	1.7 (0.8–3.5)	1/3	1.2 (0.1–11.7)		5/6	3.9 (1.0–14.8)^b^	1.8 (0.8–3.9)

^a^High grade defined by Gleason score ≥8 or 4 + 3. Industries selected had at least one significantly elevated or reduced association with prostate cancer among duration of employment categories (ever, <10 years or ≥10 years). Odds ratios estimated using multinomial models adjusted for age, first-degree family history of prostate cancer, ancestry, screening for prostate cancer, annual household income, highest level of education attained, level of physical activity, alcohol intake and body mass index

^b^The 95 % confidence interval excludes the null value. n.e.c.: not elsewhere classified

Some industries with elevated PCa risk, such as Logging industry and Protective services, provincial reflect the results from the corresponding categories for occupations, i.e. Forestry and logging occupations and Police officers and detectives, respectively. Other major industries where elevated PCa risk was found include Provincial and territorial governments (OR ever 1.5, 95 % CI 1.1–2.0) and Finance, such as banks and investment intermediaries. Positive associations were also found for employment <10 years in Urban transit systems (OR 2.4, 95 % CI 1.0–5.8) and ≥10 years in the Paper products industry (OR 2.3, 95 % CI 1.1–5.0). Elevated risk for high grade PCa was found for Provincial and territorial governments industry (OR ever 1.7, 95 % CI 1.2–2.6); in addition, elevated risk was found for employment <10 years in Local government service (OR 1.9, 95 % CI 1.1–3.4). Other industries with increased risk of high grade PCa include ever employment in Wood industries (OR 1.9, 95 % CI 1.0–3.6), Primary steel industries (OR 2.1, 95 % CI 1.0–4.5) and Gasoline service stations (OR 2.8, 95 % CI 1.4–5.5), as well as employment ≥10 years in Truck transportation (OR 2.0, 95 % CI 1.1–3.5)

Lower PCa risk was found for ever employment in Agriculture (OR 0.6, 95 % CI 0.4–0.9); Livestock combination farms was the only nested industry with a statistically significantly reduced association. Employments ≥10 years in the Aircraft (OR 0.5, 95 % CI 0.–0.8) and Air transportation (OR 0.4, 95 % 0.2–0.9) industries were associated with lower PCa risk. Employment in Food and beverage service, including restaurants, was also inversely associated with PCa.

Industries where significant positive associations with all PCa remained after SB adjustment included Logging, Chartered banks, Investment intermediaries and Provincial and territorial governments. Aside from Provincial government and Logging industries, robust associations for high grade PCa included employment ≥10 years in Truck transport (ORsb 1.7, 95 % CI 1.1–2.8) and employment <10 years for Gasoline service stations (ORsb 2.1, 95 % CI 1.2–3.7), Wood industries (ORsb 2.2, 95 % CI 1.8, 95 % CI 1.0–3.1) Local government (ORsb 1.7, 95 % CI 1.0–2.8). Inverse associations for employment in Agriculture, Aircraft and Food and beverage industries remained statistically significant after SB adjustment.

## Discussion

This study explored associations between PCa risk, including high grade tumors, and employment in a wide range of occupations and industries using data from the occupational histories of approximately 4000 subjects recruited in the general population. The major substantive findings, and how they compare with results of previous studies, are detailed in the next sections, followed by an examination of the study strengths and weaknesses.

Regarding occupations and industries associated with increased PCa risk, consistently positive associations for all and high grade PCa were found for Forestry and logging occupations, with the strongest ones (OR ≥ 1.9) found for ever employment and employment ≥10 years. Corresponding results for the Logging industry were slightly attenuated but still greater than 1.5. A recent case-control study in Northeastern Ontario found similar associations between PCa risk and employment in forestry and logging, with an OR of 2.70 (95 % CI 1.21–4.79) observed for employment ≥10 years [28]. Other results in the literature range from a weakly positive association reported in a Swedish registry study [12] to an inverse association in a large population cohort in the Nordic countries [13]. Elevated PCa risks have also been observed for other occupations related to forestry, such as forest conservationists [29] and forest law enforcement [12] although the associations found in our study mainly concerned logging. We also observed weaker associations for the wood industry as well as the pulp and papermaking occupations and industries. Elevated incidence or mortality from PCa associated with pulp and papermaking occupations or industries has been reported in some studies [12, 30]. Potential exposures associated with forestry and logging include pesticides [31], whole-body vibration [32, 33], wood dust [34], which can also be encountered in the Wood industry, and polycyclic aromatic hydrocarbons (PAHs) [35].

Police officers and detectives represent another occupational group where increased PCa risk was found throughout our study. Most of the studies recently reviewed by Wright, et al. [24] did not point towards elevated PCa incidence or mortality among police officers with the notable exception of a Dutch study which found a RR of 3.9 for the longest-held job [36]. Other studies not included in the Wright, et al. review found elevated PCa risk for these occupations [10, 37, 38], while a previous study conducted in Montreal [39] found no association between PCa and employment for the broader Protective services occupations, which also cover firefighting. For the latter, we did observe a statistically significantly increased risk of low grade PCa for employment ≥10 years (presented in Additional file 1: Table S1) which remained significant following SB adjustment. Potential exposures of police officers and detectives include PAH and non-ionizing radiation from radar guns [36, 40, 41], although the intensity of exposure associated with the use of radar guns is generally very low [42]. These occupations may also entail night-shift work, which has been associated with prostate cancer in the literature [43].

We also observed elevated PCa risk for Mixers and painting and decorating occupations, which are more directly involved with industrial chemicals. A previous case-control study in Montreal found no association between ever employment as a painter and PCa [44] although they did not make a distinction, like us, between construction and non-construction painters. Another study within the same population did find a positive association for painting, stripping and varnishing as a leisure activity [45]. Positive associations with PCa and employment in the paint and varnish industry have also been reported in another Canadian study [10]. These occupations/industries can be associated with a wide array of potential exposures, such as paints, lacquers, binding agents, pigments and solvents, as well as cadmium for which IARC considers to have limited evidence of an association with PCa [14].

There was also evidence in our study of excess PCa risk in several white collar occupations, including social sciences and administrative, management and clerical occupations. These typically entail few chemical exposures, but may reflect lower workplace physical activity levels, higher PCa screening practices, among other factors. We adjusted for these in our models with summary variables but it may be that residual confounding is at play. The literature is rather uninformative with respect to PCa risk in white collar occupations although excess risk among administrators and clerical workers has been reported [13].

We found some occupations for which elevated risks were restricted to high grade cancer, such as Bus drivers. Excess mortality from PCa for bus drivers was reported in an American study [46]. We also found elevated PCa risk for employment in the truck transport industry and, to a lesser extent, for heavy truck drivers, which have been reported in the literature, including for aggressive PCa [17, 47]. There is some evidence that whole-body vibration might play a role in the associations between driving occupations and PCa [48], but other exposures such as diesel exhaust and PAHs [39, 49], and circadian rhythm disruption [50] may be involved. Another group was Gasoline station attendants, where the elevated risk of high grade PCa was mainly found for employment <10 years. Previous evidence on this is limited; a study from 1987 observed a small, non-significant increase in mortality, based on three observed deaths [51]. Finally, the increased risk of high-grade cancer observed for employment in occupations such as gasoline station attendants, textile processing and truck drivers does not appear to reflect delayed detection for lack of screening as associations remained essentially the same when restricting the study sample to men recently screened.

Regarding Farming and agriculture, a recent meta-analysis [52] found statistically significantly increased PCa risk among farmers, while another review [53] did not find conclusive evidence of increased PCa incidence associated with employment in farming. Our findings for these occupations were generally null or even negative for employment of less than 10 years, and are comparable to those from a previous case-control study in Montreal [39]. Several factors could explain these observations. First, the study population was mainly urban, focusing on Montreal residents at recruitment, which led to a limited number of subjects previously employed in agriculture. Employment in these occupations was mainly short term (<10 years), prior to the mid-1970s, and a sizable proportion of subjects were European and Haitian immigrants. Second, employment was generally in small, family-run farms in field crop, vegetable and animal production. Chemicals thought to underlie associations with PCa in a number of previous investigations include organochlorines and organophosphates. It may well be that our study base includes fewer agricultural workers who were exposed to synthetic pesticides compared to other studies focusing on large-scale farming operations, explaining divergent findings. In addition, there is evidence that common genetic variations in xenobiotic metabolic enzymes can modulate the PCa risk associated with agricultural exposures [54], which was not accounted for in our analyses. Farmers may also be exposed to very high levels of ultraviolet radiation which may have a protective effect on PCa over several decades [55]. However, cumulative exposure in our study is not likely to be high due to the duration of employment of subjects ever employed as farmers was mostly less than 10 years. Finally, the generally lowered risks observed here might also reflect under-detection of PCa in our farmer group relative to other occupations, which was not fully captured through our consideration of screening practices.

Aside from farming, significantly reduced PCa risks were found for employment in aircraft and air transportation occupations or industries. Some studies have found some suggestive evidence of higher PCa risk among pilots [11, 46]; in our case, the subjects employed within the Air transportation minor group were split between flight deck crew and support operations (e.g. air traffic controllers), resulting in small numbers within specific occupations. Aircraft maintenance workers may also perform work outdoors. However, reviews of the job descriptions indicate that most of them appear to have worked a significant portion of the time inside hangars, limiting exposure to UV radiation.

The potential associations between employment in other occupation groups and PCa risk have been explored by several studies, but findings for most specific occupations relied on a handful of studies, several of which were quite small. Moreover, large registry-based analyses focused on PCa mortality, which does not relate well to PCa incidence, and few assessed disease aggressiveness. Reviews on the subject concluded that the evidence remains scarce, and that methodological issues hampered interpretation. Some hints were apparent for occupations entailing metal and related exposures, oil-based fluids, petroleum, PAHs, engine emissions or PCBs, but there was little or no evidence accrued for occupation groups exposed to cadmium, lead, chromium and rubber products [5, 6]. The complex mixtures encountered in many occupations render them non-specific with regard to exposures per se, re-enforcing the need to conduct studies focusing on detailed exposure assessment protocols.

The job and industry titles in this study were derived from detailed information provided by subjects about their occupations, including specific tasks. Validity studies have generally shown high concordance between historical records of employment and self-reports [56]. Assignment of occupational codes might have also entailed errors. However, the translation of the free-text job descriptions into standardized job and industry titles was conducted by experienced chemists-hygienists blinded to the subjects’ case/control status, likely minimizing inconsistencies. The coding of job titles up to the 7-digit level of the CCDO classification also provided finer employment categories than most previous studies.

Response rates in the study were relatively high for cases (79 %) and lower for controls (56 %), although these values compare reasonably well with other studies based on lengthy in-person interviews. In the event that individuals who selected themselves out of the study had characteristics related to their occupation, biased risk estimates might ensue. We compared respondents and non-respondents for several socio-economic characteristics using census information, including education, income, proportion of recent immigrants and unemployment rates; for both cases and controls, differences were minimal. In addition, ORs for the associations between African or Asian ancestry and PCa, relative to European, and for first-degree family history of PCa, were comparable to those reported in the literature. These results suggest that selection bias should not be of major concern. Our study also features several important strengths, the first being the large size of the study population comprising almost 4000 subjects, making this the largest population-based case-control study investigating the role of occupational circumstances in PCa risk. Statistical power to detect associations in the more prevalent occupational groups was excellent, although it became limited for more specific employment categories.

Second, it relied on histologically confirmed PCa cases; for controls, screening rates were very high with over 75 % reporting having been screened by PSA and/or DRE within the 2 years preceding the date of interview. In Montreal, Canada, access to the medical system is universal and free of charge. While there is no systematic PCa screening program in place, at the time of study screening was frequently integrated within the annual routine medical examination, independently of socio-economic status. Screening behavior can be influenced by a number of factors, including lifestyle, beliefs, and medical follow-ups, including those offered in the workplace, and thus can potentially confound an occupation-PCa association. The available information on PCa screening patterns in our population allowed us to adjust for screening in the models and limit the impact of disease misclassification on our results and conclusions. Moreover, analyses excluding controls not screened for PCa in the previous 2 years to limit the potential for latent PCa yielded similar findings.

Third, information on Gleason scores for cases was extracted from pathology reports and was available for nearly all subjects, allowing us to investigate potential associations stratified by PCa grade; this has generally been overlooked in studies of occupational risk factors for PCa. Fourth, the information available on recognized risk factors (e.g. ancestry, family history) and other factors (e.g. BMI, education level) associated directly or indirectly with PCa allowed us to account for these potential confounders in the inferences made.

Lastly, we used statistical methods to account for the large number of associations between employment by occupation or industry and PCa risk in order to identify the most robust results warranting further investigation. On average, 30 % of the statistically significant ORs remained as such following SB adjustment across the different analyses performed.

Little data has been accrued to date on the role of specific occupational exposures in prostate cancer risk. Agents for which there is limited evidence for humans include arsenic and inorganic arsenic compounds, cadmium and cadmium compounds, malathion, among others [14], and a role for night work is also suspected [43]. Some of these may or may not explain some of the associations observed here, but in the present population-based analysis, job and industry titles should not be viewed as reliable proxies for specific occupational exposures. Nevertheless, emerging associations can serve as leads for future work investigating specific exposures. On one hand, the identification of positive associations between employment in a given occupation or industry and PCa risk could be due to exposure to one agent, or to a diversity of agents and mixtures, not discounting the possibility of interactions between agents. On the other hand, exposure to an agent associated with PCa might occur in a range of occupations and the association might not be picked up with an analysis based on job titles, especially if the prevalence of exposure within occupations is low. Grouping subjects according to specific exposures, rather than job titles, is thus a more powerful approach to detect associations [57]. Indeed, one of the limitations identified in reviews of occupational risk factors for PCa [5, 6] has centered on the use of crude methods for exposure assessment in many studies. To this end, the translation of the occupational histories in the PROtEuS study into estimates of exposure to over 300 agents is currently ongoing.

## Conclusions

Our findings suggest excess PCa risks among forestry workers and policemen, as well as in some predominantly white collar occupations and industries such as public service, administrative and clerical work. This represents an interesting research lead as little evidence has been accrued to date on white collar occupations and cancer risk. Increased risk of high grade PCa was also found for other occupations such as gas station attendants and bus drivers that were not apparent when looking at low and high PCa grades combined. Additional investigations are needed to identify specific exposures or circumstances potentially associated with PCa among subjects employed in these occupations.

## Additional files

Additional file 1: Table S1. Associations between PCa risk (all PCa, low-grade and high-grade PCa) for ever employment and duration of employment, occupations. (XLS 702 kb)
Additional file 2: Table S3. List of occupation and industry categories featuring at least one subject ever employed and <10 subjects ever employed or 0 case or control. (XLS 449 kb)
Additional file 3: Table S2 Associations between PCa risk (all PCa, low-grade and high-grade PCa) for ever employment and duration of employment, industries. (XLS 600 kb)

## Abbreviations

AHS: Agricultural Health Study
BMI: Body-mass index
CCDO: Canadian Classification and Dictionary of Occupations
CI: Confidence interval
n.e.c.: not elsewhere classified
OR: Odds ratio
PAH: Polycyclic aromatic hydrocarbon
PCa: Prostate cancer
PSA: Prostatic specific antigen
SB: Semi-Bayes
SIC: Standardized Industrial Classification
